# Trabectedin for Patients with Advanced Soft Tissue Sarcoma: A Non-Interventional, Retrospective, Multicenter Study of the Italian Sarcoma Group

**DOI:** 10.3390/cancers13051053

**Published:** 2021-03-02

**Authors:** Emanuela Palmerini, Roberta Sanfilippo, Giovanni Grignani, Angela Buonadonna, Antonella Romanini, Giuseppe Badalamenti, Virginia Ferraresi, Bruno Vincenzi, Alessandro Comandone, Antonio Pizzolorusso, Antonella Brunello, Fabio Gelsomino, Tommaso De Pas, Toni Ibrahim, Federica Grosso, Francesca Zanelli, Maria Abbondanza Pantaleo, Laura Milesi, Libero Ciuffreda, Vittorio Ferrari, Emanuela Marchesi, Irene Quattrini, Alberto Righi, Elisabetta Setola, Elisa Carretta, Piero Picci, Stefano Ferrari

**Affiliations:** 1 Chemotherapy Unit, IRCCS Istituto Ortopedico Rizzoli, 1 Via Pupilli, 40136 Bologna, Italy; irene.quattrini@ior.it (I.Q.); elisa.carretta@ior.it (E.C.); stefanoferrari.19855@gmail.com (S.F.); 2 Department of Cancer Medicine, Fondazione IRCCS Istituto Nazionale dei Tumori, Via Giacomo Venezian, 1, 20133 Milano, Italy; Roberta.Sanfilippo@istitutotumori.mi.it; 3 Division of Medical Oncology, Candiolo Cancer Institute, FPO-IRCCS, Strada Provinciale 142, Candiolo, 10060 Torino, Italy; giovanni.grignani@ircc.it; 4 Centro di Riferimento Oncologico di Aviano (CRO Aviano), IRCCS, Via Franco Gallini 2, 33081 Aviano, Italy; abuonadonna@cro.it; 5 Azienda Ospedaliero-Universitaria Pisana, Via Roma 67, 56126 Pisa, Italy; amvromanini@gmail.com; 6 Department of Surgical, Oncological and Oral Sciences, Section of Medical Oncology, University of Palermo, Piazza Marina 61, 90133 Palermo, Italy; giuseppe.badalamenti@unipa.it; 7 IRCCS Regina Elena National Cancer Institute-Division of Medical Oncology 1-Via Elio Chianesi 53, 00144 Rome, Italy; virginia.ferraresi@ifo.gov.it; 8 Medical Oncology, University Campus Bio-Medico, Via Alvaro del Portillo 200, 00128 Rome, Italy; B.Vincenzi@unicampus.it; 9 SC Oncologia ASL Città di Torino, Ospedale San Giovanni Bosco Torino, Piazza del Donatore di Sangu, 3, 10154 Torino, Italy; alessandro.comandone@aslcittaditorino.it; 10 Istituto Nazionale Tumori IRCCS “Fondazione G. Pascale”, Via Mariano Semmola 53, 80131 Naples, Italy; a.pizzolorusso@istitutotumori.na.it; 11 Oncology 1 Unit, Istituto Oncologico Veneto IOV–IRCCS, Via Gattamelata 64, 35128 Padova, Italy; antonella.brunello@iov.veneto.it; 12 Department of Oncology and Hematology, University Hospital of Modena, Via del Pozzo 71, 41124 Modena, Italy; gelsomino.fabio@aou.mo.it; 13 Unit of Medical Oncology Sarcomas, Thymomas and Rare Tumors, European Institute of Oncology, IRCCS, Via Ripamonti 435, 20121 Milano, Italy; Tommaso.DePas@ieo.it; 14 Osteoncology and Rare Tumors Center, IRCCS Istituto Romagnolo per lo Studio dei Tumori (IRST) “Dino Amadori”, Via Piero Maroncelli 40, 47014 Meldola, Italy; toni.ibrahim@irst.emr.it; 15 Mesothelioma and Rare Cancer Unit, Azienda Ospedaliera SS. Antonio e Biagio General Hospital, Via Venezia, 16, 15121 Alessandria, Italy; federica.grosso@ospedale.al.it; 16 Dipartimento Oncologico e Tecnologie Avanzate, Arcispedale Santa Maria Nuova IRCCS Reggio Emilia, Viale Risorgimento 80, 42123 Reggio Emilia, Italy; Francesca.Zanelli@ausl.re.it; 17 Division of Oncology, IRCSS Azienda Ospedaliero-Universitaria di Bologna, Via Albertoni 15, 40138 Bologna, Italy; maria.pantaleo@unibo.it; 18 Depatement of Oncology, ASST. Papa Giovanni XXIII Hospital, Piazza OMS, 1, 24127 Bergamo, Italy; lmilesi@asst-pg23.it; 19 Medical Oncology Unit, Azienda Ospedaliero Universitaria San Giovanni Battista, Molinette, Via Santena 5, 10126 Torino, Italy; lciuffreda@cittadellasalute.to.it; 20 Dipartimento di Specialità Medico-Chirurgiche, Scienze Radiologiche e Sanità Pubblica, Oncologia Medica, Università degli Studi di Brescia, ASST Spedali Civili, Piazzale Spedali Civili 1, 25123 Brescia, Italy; vittorio.ferrari@asst-spedalicivili.it; 21 Italian Sarcoma Group Clinical Trial Unit, IRCCS Istituto Ortopedico Rizzoli, Via Pupilli 1, 40136 Bologna, Italy; emanuela.marchesi@italiansarcomagroup.org; 22 Department of Pathology, IRCCS Istituto Ortopedico Rizzoli, Via di Barbiano 1/10, 40136 Bologna, Italy; alberto.righi@ior.it (A.R.); elisabetta.setola@gmail.com or; 23 Department of Experimental, Diagnostic an Speciality Medicine, Alma Mater Studiorum, University of Bologna, Via Zamboni, 33, 40126 Bologna, Italy; 24 Laboratory of Oncologic Research, IRCCS Istituto Ortopedico Rizzoli, Via di Barbiano 1/10, 40136 Bologna, Italy; piero.picci@italiansarcomagroup.org

**Keywords:** trabectedin, soft tissue sarcoma, real-life, observational

## Abstract

**Simple Summary:**

Active therapeutic options in advanced soft tissue sarcoma (STS), able to induce durable objective responses, are scarce beyond first-line chemotherapy. Thus, new strategies and optimal sequencing in the treatment algorithm for sarcoma represents an utmost clinical challenge. This non-interventional, retrospective, multicenter study of the Italian sarcoma group aimed to provide insights of the real-world efficacy, toxicity, and management of patients with advanced STS treated with trabectedin in clinical practice across Italy. Our findings on 512 pretreated metastatic patients with multiple sarcoma histologies in terms of time-to-event outcomes (median progression-free survival of 5.1 months and median overall survival of 21.6 months) confirm the activity of this regimen in a real-life setting with a manageable and well-characterized safety profile. Our study has corroborated that in real-life clinical practice, trabectedin is mostly given as a second-line treatment to patients with a good performance status and high-grade, metastatic leiomyosarcoma and liposarcoma.

**Abstract:**

The Italian Sarcoma Group performed this retrospective analysis of patients with advanced soft tissue sarcoma, pretreated with ≥1 anthracycline-based treatment, and treated with trabectedin every three weeks. Primary endpoint was to describe real-life use of trabectedin across Italy. Secondary endpoints included objective response rate (ORR) and safety. Overall, 512 patients from 20 Italian centers were evaluated. Leiomyosarcoma (37.7%)/liposarcoma (30.3%) were the most prevalent histological types (abbreviated as L-sarcoma). Patients received a median of four trabectedin cycles (range: 1–40), mostly as a second-line treatment (~60% of patients). The ORR was 13.7% superior (*p* < 0.0001) in patients with L-sarcoma compared with patients with non-L-sarcoma (16.6% vs. 9.0%). Median progression-free survival (PFS) was 5.1 months, whereas median overall survival (OS) was 21.6 months. Significantly better PFS and OS were observed in patients with L-sarcoma, those with objective responses and/or disease stabilization, treated in an early line and treated with reduced dose. Bone marrow toxicity (61.4%) and transaminase increases (21.9%) were the most common grade 3/4 adverse events. The results of this real-life study suggest that trabectedin is an active treatment, which is mostly given as a second-line treatment to patients with a good performance status and high-grade, metastatic L-sarcoma (clinical trial information: NCT02793050).

## 1. Introduction

Soft tissue sarcomas (STS) are a heterogeneous group of more than 100 different mesenchymal malignancies arising from extraskeletal connective tissues, which represent less than 2% of all adult tumors worldwide [1,2]. Despite radical en-bloc resection with curative intent, up to 30% of patients with localized sarcoma ultimately experience metastatic relapse at distant sites. Patients with advanced or metastatic sarcoma carry a poor prognosis, with an estimated median survival of approximately 1 year from the start of first-line anticancer therapy [3]. In patients with advanced disease, the optimum first-line treatment involves palliative systemic chemotherapy that has been unchanged for four decades and still consists of anthracycline-based chemotherapy [4]. After failure or intolerance of conventional front-line chemotherapy, the optimal sequencing of second-line options in the treatment algorithm for STS has not been well defined [5]. According to the European Society for Medical Oncology—European Reference Network for rare adult solid cancers (ESMO-EURACAN) Clinical Practice Guidelines, trabectedin is recommended for second- or beyond-line treatment for advanced STS after failure of anthracycline-based chemotherapy [2].

Trabectedin (Yondelis^®^, PharmaMar, Madrid, Spain) is a semi-synthetic drug originally isolated from the sea squirt Ecteinascidia turbinata. Trabectedin is a DNA-binding agent with a complex pleiotropic mechanism of action which, besides to induced direct growth inhibition and death of malignant cells, selectively modulates inflammatory responses in the tumor microenvironment and inhibits the factors that promote tumor growth, angiogenesis, and metastasis [6,7,8]. Trabectedin was the first marine-derived antineoplastic drug approved in 2007 in the European Union and in many other countries around the globe for the treatment of patients with advanced STS after failure of anthracyclines and ifosfamide, or who are unsuited to receive these agents [9]. Based on the results of a pivotal, randomized, active-controlled phase III study that evaluated the efficacy and safety of trabectedin as compared with dacarbazine, in 2015 trabectedin was also approved by the U.S. Food and Drug Administration for patients with advanced liposarcoma or leiomyosarcoma (commonly abbreviated as L-sarcomas) after failure of prior anthracycline-containing chemotherapy [10,11]. Trabectedin treatment is also feasible in non-L-sarcomas as it has demonstrated efficacy in patients with a variety of histologically different sarcoma subtypes [12,13]. Particularly, in patients with translocation-related sarcoma unresponsive or intolerant to standard chemotherapy regimens the treatment with trabectedin significantly reduced the risk of disease progression and death as compared with best supportive care [14]. In addition, numerous compassionate expanded access programs (EAPs) [3,15] as well as a retrospective [16] and a real-life prospective, non-interventional studies with trabectedin [17] have consistently supported that trabectedin confers clinically meaningful long-term benefits to patients with multiple STS histotypes.

Herein, we have carried out an observational retrospective study of trabectedin (Trabectedin in Soft Tissue Sarcomas: A Retrospective Observational Analysis; TrObs trial) in patients with advanced STS, treated with trabectedin within the Italian Sarcoma Group centers (clinical trial information: NCT02793050). Our primary goal was to obtain insights about the use of trabectedin and its efficacy and safety in routine clinical practice across Italy, acquired from a more diverse and often underrepresented patient population than that recruited in clinical trials.

## 2. Materials and Methods

The aim of this non-interventional, retrospective, multicenter TrObs study was to evaluate the treatment outcomes as assessed in routine clinical practice in patients with advanced STS across Italy. Patients were treated with trabectedin in accordance with the marketing authorization and local clinical practice. As per non-interventional nature of the study, there was no involvement with any treatment decision for the patients included in the study and no additional per protocol diagnostic or therapeutic measures were performed during the study.

The primary endpoint was to describe the clinical characteristics of patients treated with trabectedin according to the approved prescribing indication. Secondary endpoints included objective response rate (ORR) according to treating physician evaluation based on the Response Evaluation Criteria in Solid Tumors (RECIST) version 1.1 [18], the disease control rate (DCR), defined as the percentage of patients with a radiological complete response (CR) or partial response (PR) and/or stable disease (SD), and an evaluation of applied doses and treatment discontinuations. The secondary endpoints also included the assessment of progression-free survival (PFS), overall survival (OS), and their fixed-time estimations. Finally, as exploratory objective we also assessed the relation between tumor response with prior chemotherapy lines, pattern of disease, and histotype.

All study procedures were carried out in accordance with the Declaration of Helsinki and its later amendments and local regulations on clinical trials, and were approved by the institutional review boards of each participating center. Due to the de-identified nature of the data collected in this study, signed informed consents were obtained from all alive study participants at enrolment.

Eligible patients were adults who received trabectedin according to the approved indication. Patients enrolled in clinical trials with trabectedin were excluded. Trabectedin was administered in accordance with the marketing authorization and the treating clinician’s discretion depending on the patient’s conditions and previous chemotherapy. The recommended dose of trabectedin for the treatment of STS is 1.5 mg/m^2^ body surface area, administered as an intravenous infusion over 24 h with a 3-week interval between cycles. There were no predefined limits to the number of administered trabectedin cycles. The treatment could be modified at clinician’s discretion depending on the patient’s condition, toxicities, and previous chemotherapy. Pretreatment with corticosteroids and/or additional antiemetics were administered in accordance with local clinical practice. After the treatment with trabectedin, patients could have been treated with subsequent anticancer therapies or supportive care according to the treating clinician’s best clinical judgment.

The study period for data collection corresponded to patients’ treatment period, which spans from the first trabectedin dose until patient discontinuation for any reason or the patient’s death, whichever comes first. According to the reimbursement rules, the response evaluation was first performed after two cycles of treatment. The results of imaging and response evaluations were assessed by local investigators according to their routine clinical practice. Adverse events (AEs) were classified according to Common Terminology Criteria for Adverse Events (CTCAE) v.4.0. Only grade 3/4 AEs were collected during the study.

Descriptive analysis was performed with appropriate statistical methods (i.e., median, minimum, and maximum for continuous variables; numbers and percentages for categorical variables). The primary analyses assessing efficacy and safety were carried out in all enrolled patients who received at least one dose of trabectedin. PFS and OS and their fixed-time estimations were estimated according to the Kaplan–Meier method and were compared using the log-rank test. All *p* values were descriptive in nature and the significance level selected was 0.05. The PFS and OS analyses were defined as the time interval from the first administration of trabectedin to the earliest date of disease progression or death, regardless of cause (whichever occurred first) for PFS, whereas OS was defined as the time between the start of trabectedin and patient death from any cause. Patients considered lost to follow-up, with no reported disease progression, and alive, were censored at the day of the last visit. Multivariate survival analysis with the variables that proved to be significant in univariate analysis was performed using Cox regression model.

## 3. Results

### 3.1. Patient Characteristics

From January 2010 to December 2015 a total of 512 patients (294 women, 57.4%) with high-grade sarcoma and from 20 Italian recruiting sites were included in the analysis set (Table 1). Patients had a median age of 57.0 years (range: 20–87) and an Eastern Cooperative Oncology Group (ECOG) performance status score of 0/1 was recorded in 469 patients (91.6%). Central review of sarcoma diagnosis was performed in 188 patients (36.7%). Most patients had leiomyosarcoma (*n* = 193, 37.7%), 117 of whom had non-uterine leiomyosarcoma (22.9%), followed by liposarcoma (*n* = 155, 30.3%), undifferentiated pleomorphic sarcoma (*n* = 45, 8.8%), and synovial sarcoma (SyS; *n* = 40, 7.8%). Among patients with liposarcoma, 53 patients had myxoid round cell liposarcoma (MRCL, 10.4%), whereas 50 (9.8%) and 15 (2.9%) patients had dedifferentiated liposarcoma (DDL) and pleomorphic liposarcoma, respectively. The majority of patients had metastatic disease (*n* = 442, 86.4%), mostly being bilateral lung metastases (*n* = 226, 51.1%). Overall, 433 patients (84.6%) had undergone cytoreductive surgery, 272 (62.8%) of whom had surgically free disease. Patients were pretreated with a median of one prior chemotherapy line (range: 1–5), 59.4% of whom received prior anthracycline-based chemotherapy.

A total of 290 (62.5%) out of 465 patients with available data at follow up, received a subsequent antineoplastic treatment, mostly being pazopanib (*n* = 72, 24.8%), dacarbazine (*n* = 56, 19.3%), ifosfamide (*n* = 41, 14.1%), gemcitabine monotherapy (*n* = 37, 12.8%), or in combination with docetaxel (*n* = 23, 7.9%) and doxorubicin (*n* = 18, 6.2%).

### 3.2. Treatment Exposure

Patients received a median of four trabectedin cycles (range: 1–40 cycles), with 187 (36.5%) patients receiving ≥six cycles over a median treatment duration of 2 months (range: 1–47) (Table 2). Trabectedin was given either at a reduced starting dose of 1.3 mg/m^2^ (*n* = 309, 60.3%) or at the standard dose of 1.5 mg/m^2^ (*n* = 177, 34.6%), while in seven patients the dose was not specified (1.4%). Trabectedin was mostly given as a second- line of treatment (*n* = 304, 59.4%). Dose reductions during the study occurred in 100 of patients, commonly due to hematological toxicity in 44 patients followed by hepatic toxicity in 21 and asthenia in 17 patients. All patients received steroid premedication as per each institution practice.

Among 399 patients (77.9%) who discontinued the treatment the most common cause was disease progression (*n* = 309, 77.4%), followed by treatment-related toxicity in 30 (7.5%), patients’ choice and worsening of clinical conditions in 16 patients each (4.0%), and complete surgery remission in 12 patients (3.0%).

### 3.3. Efficacy

**Figure 1:**
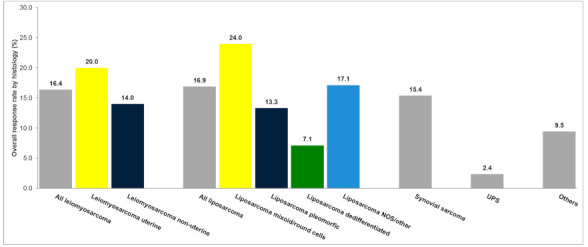
Overall response rate by histology as per RECIST v.1.1. UPS, Undifferentiated pleomorphic sarcoma.

Overall, seven patients (1.4%) had a CR, and 63 (12.3%) patients achieved a PR, reaching the ORR of 13.7%. Additionally, 169 patients (33.0%) had SD as a best result for a DCR of 46.7% (Table 3). The median time from trabectedin treatment to best response was 2.2 months (range: 0.2–73). The ORR was statistically superior (*p* < 0.0001) in patients with L-sarcoma (16.6%) than in patients with non-L-sarcoma (9.0%). In particular, patients with MRCS, SyS, and DDL obtained an ORR of 24.0%, 15.4%, and 7.1%, respectively (Figure 1). In contrast, no statistically significant (*p* = 0.58) differences in ORR were observed between patients treated as second- (15.5%), third- (12.4%) or ≥ fourth-line (10.2%) of treatment, according to the starting dose of trabectedin (standard: 17.3% vs. reduced dose of 1.3 mg/m^2^: 13.2%; *p* = 0.39), nor by the disease metastatic pattern comparing the patients with lung-only vs. non lung-only metastases at baseline (12.5% vs. 16.0; *p* = 0.2).

**Figure 2:**
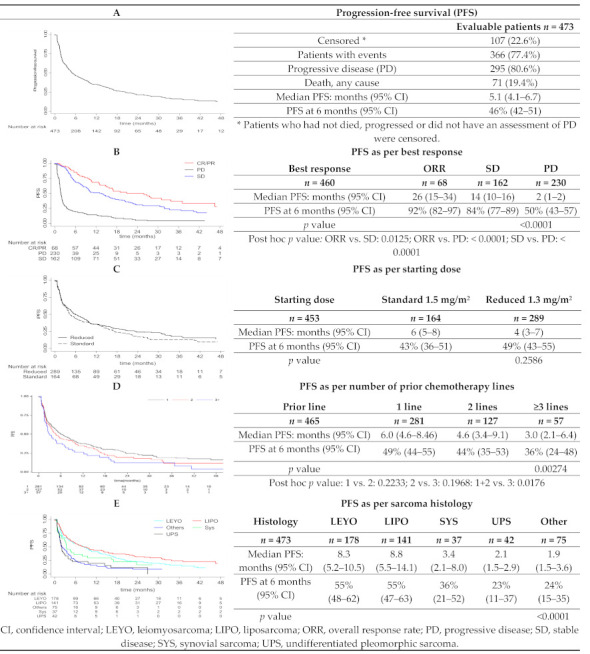
Kaplan–Meier plots of progression-free survival and univariate analyses.

Median PFS was 5.1 months (95% CI: 4.1–6.7) with 46% (95% CI: 42–51) of patients free from progression at 6 months after treatment (Figure 2A). Significantly longer median PFS and higher PFS rates at 6-months were observed in patients who obtained objective responses or SD (*p* < 0.001; Figure 2B), those who received trabectedin as second-line or third-line treatment compared to patients who received trabectedin as ≥fourth-line treatment (*p* < 0.00274; Figure 2D) and those with L-sarcoma (*p* < 0.0001; Figure 2E). No statistical differences were observed according to the starting dose of trabectedin (*p* = 0.26, Figure 2C), patients’ age (*p* = 0.95), and pattern of metastases (*p* = 0.50).

**Figure 3:**
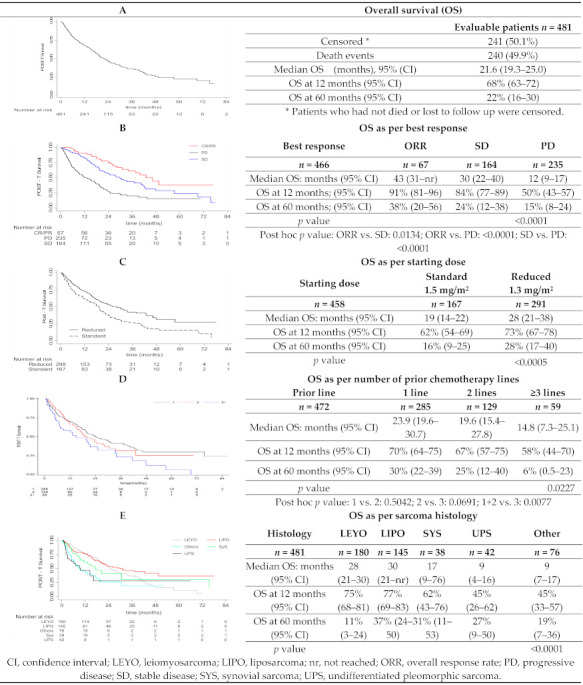
Kaplan–Meier plots of overall survival and univariate analyses.

After a median follow-up of 24.5 months (95% CI: 22.7–28.7) median OS was 21.6 months (95% CI: 19.3–25.0) with 68% (95% CI: 63–72) and 22% (95% CI: 16–30) of patients alive 12 and 60 months after treatment, respectively (Figure 3A). Statistically larger median OS and OS rates at 12 and 60 months were also observed in patients who obtained objective response or SD (*p* < 0.001; Figure 3B), those treated with reduced starting dose of trabectedin (*p* < 0.0005; Figure 3C), in patients who received trabectedin as an early treatment compared to patients with more extensive prior therapy (*p* < 0.00227; Figure 3D), and patients with L-sarcoma (*p* < 0.0001; Figure 3E). No statistical differences in OS were observed according to patients’ age (*p* = 0.11) and pattern of metastases (*p* = 0.09).

A multivariate analysis of variables found to be significant in the univariate analyses of OS also revealed to be significant prognostic factors associated with longer OS (i.e., reduced trabectedin dose, L-sarcoma, objective response and treated early with trabectedin) (Table 4).

### 3.4. Safety

Overall, 114 out of 493 patients (23.1%) with available data (19 patients had missing data) had at least one grade 3/4 AE, 56.4% of whom were treated with a standard dose and 43.6% with a reduced dose (i.e., 1.3 mg/m^2^ or total dose 2.6 mg) of trabectedin (*p* = 0.0001). Most common grade 3/4 AEs seen with trabectedin were bone marrow toxicity (*n* = 70, 61.4%) and transaminase increase (*n* = 25, 21.9%). Again, grade 3/4 AEs were statistically more common (*p* < 0.0001) in patients treated with the standard starting dose (36.5%) as compared with those who received a reduced starting dose of trabectedin (15.4%).

## 4. Discussion

The TrObs study was conducted as a retrospective analysis of an unselected heterogeneous population with recurrent STS and treated with trabectedin. The principal aim was to provide an overview of the patients’ characteristics and outcomes in routine real-life clinical practice across Italy. Acknowledging that the results of TrObs study cannot be considered representative of the whole group of STS patients treated in Italy, they surely provide useful insights in the Italian real-life clinical practice, especially considering that we included data from 512 patients in 20 referral centers.

Overall, the results of this retrospective analysis consistently confirm that trabectedin is an active treatment that provides clinically meaningful benefits to patients with advanced sarcoma of multiple histologies. In the present study, reported median PFS (5.1 months), OS (21.6 months), and ORR (13.7%) compare favorably with the outcomes of the previous pivotal phase II/III clinical study reports, in which only patients with L-sarcomas were included [9,10] (Table 5). The efficacy outcomes from TrObs study are especially encouraging as it is more common to see a reduced activity when a regimen is given to the general population outside of a clinical trial. Concerning other sarcoma histotypes, our results suggest that trabectedin might be offered to patients with synovial sarcoma who obtained meaningful ORR (15.4%) and median PFS (3.4 months) with a PFS rate at 6 months of 46%. Those data are comparable to the results of a multicenter, European, retrospective study in patients with synovial sarcoma that reported an ORR of 15%, and median PFS of 3 months with 23% of patients free from progression at 6 months after treatment [19].

On the other hand, ORR was limited in patients with UPS (2.1%). Moreover, our results also confirm that liposarcoma subclassification is relevant, with ORR ranging from 7.1% in dedifferentiated liposarcoma to >24% in MRCL. These results underscore the importance of accurate histopathological classification to identify each sarcoma entity [20]. These differences should be taken into account particularly in those patients with oligometastatic and potentially resectable disease.

The results of this study compare well with those of two large compassionate expanded access programs (EAPs) [3,15]. We should point out that our patients were treated from 2010 to 2015, whereas in the two EAPs the patient data were collected from 2003–2008 [15] and 2005–2010 [3]. Arguably, advances in supportive care could explain, at least partly, the improved outcomes in our series. Our findings are in line with the results of a large retrospective analysis of the RetrospectYon database [16] and a real-life prospective, non-interventional Y-IMAGE study [17] (Table 5). Albeit heterogeneous, demographic and disease characteristics in all three studies were quite comparable. Following an appraisal of baseline clinical characteristics from RetrospectYon and Y-IMAGE with those from TrObs study, we noted some differences that could be suggestive of different prognoses. For instance, the number of patients with liposarcoma was higher in TrObs (30.3%) compared with RetrospectYon (18.2%) and Y-IMAGE studies (23.4%). In TrObs trial, the number of patients who had retroperitoneal sarcoma was higher than that observed in RetrospectYon (51.8% vs. 20.7%). Retroperitoneal sarcoma was associated with improved survival, possibly because of the higher prevalence of low-grade cases and loco-regional sarcomas, mostly being liposarcomas [15,21]. Importantly, 100% of the patients included in TrObs study had a high-grade sarcoma compared with 55.5% of patients in Y-IMAGE and 51.4% of patients in RetrospectYon. In addition, in TrObs more patients received trabectedin as second-line treatment (59.4%), and none in first line, as compared with the number of patients from Y-IMAGE treated both in the first- (10.1%) and second-line (39.9%) setting. Moreover, in TrObs more patients with a good performance status with ECOG score of 0/1 were recruited (91.6%), as compared with RetrospectYon and Y-IMAGE (ECOG score 0/1: 73.7% and 70.6%, respectively). Finally, in TrObs less patients were pretreated with prior radiotherapy (33.4%) than in Y-IMAGE (53.7%), whereas comparable numbers of patients of patients in TrObs (84.6%), RetrospectYon (85.3%), and Y-IMAGE (91.3%) had previously undergone radical surgery. In spite of these differences, median PFS in the current series is comparable to those observed in RetrospectYon and Y-IMAGE studies (Table 5), with a 6-month PFS rate of 46% in all sarcoma histotypes (L-sarcomas: 55%; non-L-sarcomas: 26%) that largely exceed the 6-month PFS rate threshold of 14% established by the EORTC for active drugs for the treatment of unselected STS [21].

In line with previously published data from a pooled analysis of five phase II trials, which assessed the effect of age on the efficacy and safety of trabectedin [22], we also observed no statistical difference in PFS and OS between patients younger than 60 years and elderly patients, confirming that trabectedin in real-life setting is a feasible treatment regardless of patient age. Not surprisingly, the patients with an objective response and/or SD obtained significantly longer PFS and OS in comparison with those who progressed during the treatment. Similar to other findings [21], our analysis did not observe statistically significant difference in PFS and OS according to the pattern of metastases (i.e., lung vs. other metastases). For the treatment of advanced STS, the recommended dose of trabectedin is 1.5 mg/m^2^ body surface area, administered as an intravenous infusion over 24 hours with a three-week interval between cycles. Nevertheless, a dose of 1.3 mg/m^2^ every three weeks or a dose of 0.58 mg/m^2^ 3-hour weekly infusion for 3 weeks every 4 weeks, have has been shown to be feasible, with encouraging results in STS [9,23]. In our study, we observed a statistically significant larger OS in patients treated with a reduced dose of trabectedin at 1.3 mg/m^2^, with no difference in ORR and PFS.

A post-hoc analysis of patients’ characteristics evidenced that among patients treated with trabectedin at the dose of 1.3 mg/m^2^ as compared with those treated at 1.5 mg/m^2^, there were more patients with ECOG performance status score of 0 (69.3% vs. 49.2%), diagnosed with liposarcoma (30.7% vs. 26.6%), pretreated with ≥2 previous lines (44.0% vs. 33.9%), and treated in a high-load referral center (>30 patients/center in the present series, 87.4% vs. 68.9%). Moreover, although treatment discontinuation was greater in patients treated with 1.5 mg/m^2^ (85.9% vs. 75.1%) disease progression was the cause of discontinuation most frequently in the 1.3 mg/m^2^ group (80.1% vs. 74.5%). Conversely, other larger studies reported superior disease control following the treatment with trabectedin under the recommended schedule, which we believe should remain the standard treatment in the second-line setting for patients with advanced or metastatic STS [9,10].

The safety of trabectedin was lined up with prior experience and reports reflecting the well-characterized myelosuppression and transaminase increases [24]. No drug-related deaths or new or unexpected AEs were observed. In our study, more than a third of the patients received ≥six cycles of trabectedin, suggesting an acceptable safety profile that allowed lengthened treatment with trabectedin (i.e., up to 40 cycles). This is consistent with previous reports where comparable or even higher numbers of patients were treated with ≥six cycles (e.g., RetrospectYon: 34.4% and Y-IMAGE: 56.9% of patients) [15,16,17,25].

It is well known that treatment duration is a crucial factor for long-term outcomes with trabectedin. The significant differences in PFS and OS, observed between long-term treatment and treatment discontinuation strategies (i.e., treatment interruption after six cycles), was demonstrated by retrospective [15,16] and prospective series [26]. In particular, the prospective controlled trial T-DIS reported that the therapeutic benefit of treatment maintenance with trabectedin until disease progression or treatment intolerance compared to those who interrupted the treatment after six treatment cycles was associated with improved median PFS (7.2 vs. 4.0 months, *p* = 0.02) and median OS (27.9 vs. 16.5 months, *p* = 0.12) [26]. Therefore, according with the terms of the marketing authorization of trabectedin, there are no pre-defined limits to the number of cycles administered, with patients receiving up to 40 cycles in the present study.

## 5. Conclusions

In conclusion, the findings of this non-interventional, multicenter, retrospective study suggest that trabectedin in real-life clinical practice in Italy is mostly given as a second-line treatment to patients with high-grade, metastatic L-sarcoma and with a good performance status. Our data further support that trabectedin is a clinically meaningful, safe, and long-term option for pre-treated patients with multiple sarcoma histologies. Differences in ORR by sarcoma histotype might help to define optimal strategy in the contest of oligometastatic disease.

## Figures and Tables

**Table 1:** Patient and disease characteristics at baseline.

Patients (*n*)	Enrolled Patients; *n* = 512	
Age at study entry (years); *n* = 505	Median	57.0
	Range (min–max)	20.0–87.0
Age group	≤60	288 (56.2%)
	>60	217 (42.4%)
	Missing	7 (1.4%)
Gender	Male	218 (42.6%)
	Female	294 (57.4%)
Histology	Leiomyosarcoma (LEYO)	193 (37.7%)
	LEYO non-uterine	117 (22.9%)
	LEYO uterine	76 (14.8%)
	Liposarcoma (LPS)	155 (30.3%)
	LPS myxoid-round cells	53 (10.4%)
	Dedifferentiated LPS	50 (9.8%)
	Pleomorphic LPS	15 (2.9%)
	Undifferentiated pleomorphic sarcoma	45 (8.8%)
	Synovial sarcoma	40 (7.8%)
	Myxofibrosarcoma	13 (2.5%)
	Solitary fibrous tumor	11 (2.2%)
	Spindle cells non otherwise specified	11 (2.1%)
	Other *	44 (8.6%)
Site of primary tumor at first diagnosis	Retroperitoneal	265 (51.8%)
	Extremity	157 (30.7%)
	Trunk	38 (7.4%)
	Other **	30 (5.8%)
	Missing	22 (4.3%)
Eastern Cooperative Oncology Group (ECOG) performance status	0	321 (62.7%)
	1	148 (28.9%)
	2	22 (4.3%)
	3	1 (0.2%)
	Missing	20 (3.9%)
Tumor stage at study entry	Locally advanced	67 (13.1%)
	Metastatic ***	406 (79.3%)
	Lung metastases	289 (56.4%)
	Bone metastases	102 (20.0%)
	Other metastases	285 (55.7%)
	Both	36 (7.0%)
	Missing	3 (0.6%)
Prior treatments	Prior surgery Yes No	433 (84.6%) 79 (15.4%)
	Prior radio therapy Yes No Missing	171 (33.4%) 314 (61.3%) 27 (5.3%)
	Prior chemotherapy (*N* = 503) Yes No Missing	503 (98.2%) 0 9 (1.8%)
No. of lines of prior chemotherapy; *n* = 503	Median	1.0
	Range (Min–Max)	1–5
No. of lines of prior chemotherapy	1 line	304 (59.4%)
	2 lines	139 (27.1%)
	≥3 lines	60 (11.7%)
	Missing	9 (1.8%)

Data shown are numbers and percentages or median and range values of patients with available data. * Other histological types of sarcoma are listed in Table S1. ** Other primary sites included thoracic (lung and non-lung), head and neck, skin, perianal sites. *** Those figures include the patients with metastases (*n* = 406) and both locally advanced and metastatic disease (*n* = 36).

**Table 2:** Trabectedin exposure.

Treatment Delivery (*n*)	Treated Patients; *n* = 512	
Starting dose	1.3 mg/m^2^	309 (60.3%)
	1.5 mg/m^2^	177 (34.6%)
	Top dose 2.6 mg	7 (1.4%)
	Missing	19 (3.7%)
Time on treatment (months)	Median (range)	2 (1–47)
Cycles per patient from the study enrollment	Median (range), *n* = 505	4 (1–40)
	<6 cycles	318 (62.1%)
	≥6 cycles	187 (36.5%)
	Missing	7 (1.4%)

Data shown are numbers and percentages or median and range values.

**Table 3:** Response assessment of trabectedin.

Best Response According to Physician Evaluation RECIST v.1.1	*n* = 512
Complete response (CR)	7 (1.4%)
Partial response (PR)	63 (12.3%)
Stable disease (SD)	169 (33.0%)
Progressive disease (PD)	253 (49.4%)
Not available	20 (3.9%)
Objective response rate (ORR; CR + PR); 95% Confidence interval (CI)	70 (13.7%); 95% CI: 11.2–17.2
Disease control rate (DCR; ORR + SD); 95% Confidence interval (CI)	239 (46.7%); 95% CI: 43.2–51.9

**Table 4:** Multivariate analysis of overall survival.

Multivariate Analysis *		Overall Survival	
		HR (95% CI)	Pr > Chi Square
Starting dose	Standard vs. reduced	1.58 (1.19–2.08)	0.0013
Histology	Non-L-sarcoma vs. L-sarcoma	1.64 (1.21–2.21)	0.0014
Response to trabectedin	PD vs. ORR	3.39 (2.14–5.36)	<0.0001
	SD vs. ORR	1.66 (1.04–2.66)	
Lines of prior chemotherapy	2 lines vs. 1 line	1.31 (0.95–1.83)	0.0175
	≥3 lines vs. 1 line	1.68 (1.15–2.45)	

* Multivariate analysis included only the variables that proved to be significant in univariate analyses using the following prognostic factors: starting dose of trabectedin, sarcoma histology, response to trabectedin, number of prior lines of chemotherapy, age, and pattern of metastases (lung vs. non-lung). CI, confidence interval; HR, hazard ratio; ORR, overall response rate; PD, progressive disease; SD, stable disease.

**Table 5:** Relevance of the TrObs results within the context of trabectedin treatment for recurrent advanced STS.

Median (95% CI)	Advanced Sarcoma	PFS (months)	PFS-6 (%)	OS (months)	ORR (%)	SD (%)	DCR (%)
**Pivotal Clinical Trials**							
Demetri G et al. (STS-201) (Trabectedin 24-h arm) [9]	L-sarcoma; *n* = 136	3.3 (2.1*–*4.6)	37	13.9 (12.5*–*18.6)	5.6 (2.3*–*11.2)	52.8	58.4 (49.3*–*67.2)
Demetri G et al. (SAR-3007) (Trabectedin arm) [10]	L-sarcoma; *n* = 345	4.2	37	13.7 (12.2*–*16)	9.9 (6.9*–*13.5)	51	61.2
**Expanded Access Programs**							
Worldwide expanded access program Samuels et al. [3]	STS; *n* = 807	NA	NA	11.9	5.9 (4.4*–*7.8)	43	48.5
	L-sarcoma; *n* = 476	NA	NA	16.2 (14.1*–*19.5)	6.9 (4.8*–*9.6)	47	54.2
	non-L-sarcoma; *n* = 302	NA	NA	8.4 (7.1–10.7)	4.0 (2.1*–*6.8)	33.8	37.7
French ATU compassionate use program, Blay et al. [15]	STS; *n* = 181	3.6	39	16.1	10.0	39.0	49.0
**Prospective, Non-Interventional Study**							
Y-IMAGE study Buonadonna, A et al. [17]	STS; *n* = 218	5.9 (4.9–7.8)	49	21.3 (18.8–24.3)	26.6 (20.9–33)	39	65.6 (58.9–71.9)
**Retrospective, Non-Interventional Studies**							
French Retrospect Yon database Le Cesne et al. [16]	L-sarcoma; *n* = 481	5.7 (4.9–6.5)	NA	15.0 (13.2–16.8)	18.6	54	72.6
	STS; *n* = 804	4.4 (3.9–4.9)	40	12.2 (11.0–13.3)	16.5	50.1	66.7
TrObs study Palmerini et al.	STS; *n* = 512	5.1 (4.1–6.7)	46	21.6 (19.3–25.0)	13.7	33.0	46.7
	L-sarcoma; *n* = 348	8.3 (6–10.1)	55	25.9 (22.4–33.4)	16.1	37.4	53.4
	non-L-sarcoma; *n* = 164	2.4 (1.8–3.4)	26	11.3 (8.1–16.3)	8.5	23.8	32.3

Results of time-to-event endpoints are median and 95% confidence intervals with available or reported data. CI; confidence intervals; DCR, disease control rate; h, hour; L-sarcoma, liposarcoma or leiomyosarcoma; NA, not available; NR, not reached; ORR, objective response rate; OS, overall survival; PFS, progression-free survival; PFS-3/-6, PFS rate at 3/6 months; SD, stable disease; STS, soft tissue sarcoma; TrObs, Trabectedin in Soft Tissue Sarcomas: A Retrospective Observational Analysis; TRS, translocation-related sarcoma.

## Data Availability

The data presented in this study are available in the article. However, de-identified individual data might be made available following publication by reasonable request and on a case-by-case basis to the corresponding author, including the Clinical Study Results and statistical analysis plan. A research proposal should be included, which will be evaluated by the Italian Sarcoma Group and the ethics committee for clinical investigation.

## Supplementary Materials

The following are available online at https://www.mdpi.com/2072-6694/13/5/1053/s1, Table S1: Other sarcoma histologies (*n* = 44).
