# Supplementary material for: Trabectedin for Patients with Advanced Soft Tissue Sarcoma: A Non-Interventional, Retrospective, Multicenter Study of the Italian Sarcoma Group
Source: Cancers (Basel). 2021 Mar 2;13(5):1053. doi: 10.3390/cancers13051053 (PMC7958606; doi:10.3390/cancers13051053)
Supplement: Supplementary file 1 [file cancers-13-01053-s001.pdf]

cancers

MDPI

Trabectedin for Patients with Advanced Soft Tissue Sarcoma: A Non-Interventional, Retrospective, Multicenter Study of the Italian Sarcoma Group

Emanuela Palmerini, Roberta Sanfilippo, Giovanni Grignani, Angela Buonadonna, Antonella Romanini, Giuseppe Badalamenti, Virginia Ferraresi, Bruno Vincenzi, Alessandro Comandone, Antonio Pizzolorusso , Antonella Brunello, Fabio Gelsomino, Tommaso De Pas, Toni Ibrahim, Federica Grosso, Francesca Zanelli, Maria Abbondanza Pantaleo, Laura Milesi, Libero Ciuffreda, Vittorio Ferrari, Emanuela Marchesi, Irene Quattrini, Alberto Righi, Elisabetta Setola, Elisa Carretta, Piero Picci and Stefano Ferrari

Table S1. Other sarcoma histologies (n=44).

Other histologies (n=44)	Response (n)				Total
	CR/PR	PD	SD	n.a.	
Alveolar soft part sarcoma	1	.	1		2
Clear cell sarcoma	.	2	.		2
Desmoplastic small round cell tumor	1	2	.		3
Dermatofibrosarcoma protuberans				1	1
Epithelioid sarcoma	.	2	3		5
Extraskeletal myxoid chondrosarcoma	.	.	1		1
Malignant fibrous histiocytoma	1	1	1		3
Malignant peripheral nerve sheath tumor	.	2	1		3
Mesenchymal sarcoma	.	2	1	1	4
Myoepithelioma	.	.	1		1
Para-articular malignant schwannoma	.	1	.		1
Rhabdomyosarcoma	.	2	.		2
Epithelioid leiomyosarcoma anaplastic	.	1	.		1
Extra skeletal mesenchymal chondrosarcoma	.	.	1		1
Hemangioendothelioma	.	1	.		1
Malignancy small cells	.	1	.		1
Myxoid chondrosarcoma	.	1	.		1
Monomorphic endometrial undifferentiated sarcoma				1	1
High-grade stromal sarcoma	1	.	.		1
High-grade endometrial stromal sarcoma	.	1	.		1
Sarcoma with myogenic differentiation				1	1
Soft tissue sarcoma NOS	.	2	.		2
High grade undifferentiated endometrial sarcoma	.	.	1		1
Uterine undifferentiated sarcoma	.	.	1		1
Uterine adenosarcoma NOS	.	1	.	1	2
Uterine sarcoma NOS	.	1	.		1
**Total**	4	23	12	5	44

CR, complete response; n.a., not available; NOS, not otherwise specified; PD, progressive disease; PR, partial response; SD, stable disease.
